# Transiency of retinal ganglion cell action potential responses determined by PSTH time constant

**DOI:** 10.1371/journal.pone.0183436

**Published:** 2017-09-12

**Authors:** Alma Ganczer, Márton Balogh, László Albert, Gábor Debertin, Tamás Kovács-Öller, Béla Völgyi

**Affiliations:** 1 MTA-PTE NAP B Retinal Electrical Synapses Research Group, Pécs, Hungary; 2 Department of Experimental Zoology and Neurobiology, University of Pécs, Pécs, Hungary; 3 János Szentágothai Research Center, University of Pécs, Pécs, Hungary; National Eye Centre, UNITED STATES

## Abstract

Retinal ganglion cells (RGC) have been described to react to light stimuli either by producing short bursts of spikes or by maintaining a longer, continuous train of action potentials. Fast, quickly decaying responses are considered to be transient in nature and encode information about movement and direction, while cell responses that show a slow, drawn-out response fall into the sustained category and are thought to be responsible for carrying information related to color and contrast. Multiple approaches have been introduced thus far to measure and determine response transiency. In this study, we adopted and slightly modified a method described by Zeck and Masland to characterize RGC response transiency values and compare them to those obtained by alternative methods. As the first step, RGC spike responses were elicited by light stimulation and peristimulus time histograms (PSTHs) were generated. PSTHs then were used to calculate the time constant (PSTHτ approach). We show that this method is comparable to or more reliable than alternative approaches to describe the temporal characteristics of RGC light responses. In addition, we also show that PSTHτ-s are compatible with time constants measured on RGC and/or bipolar cell graded potentials; thus they are suitable for studying signaling through parallel retinal pathways.

## Introduction

**Fig 1:**
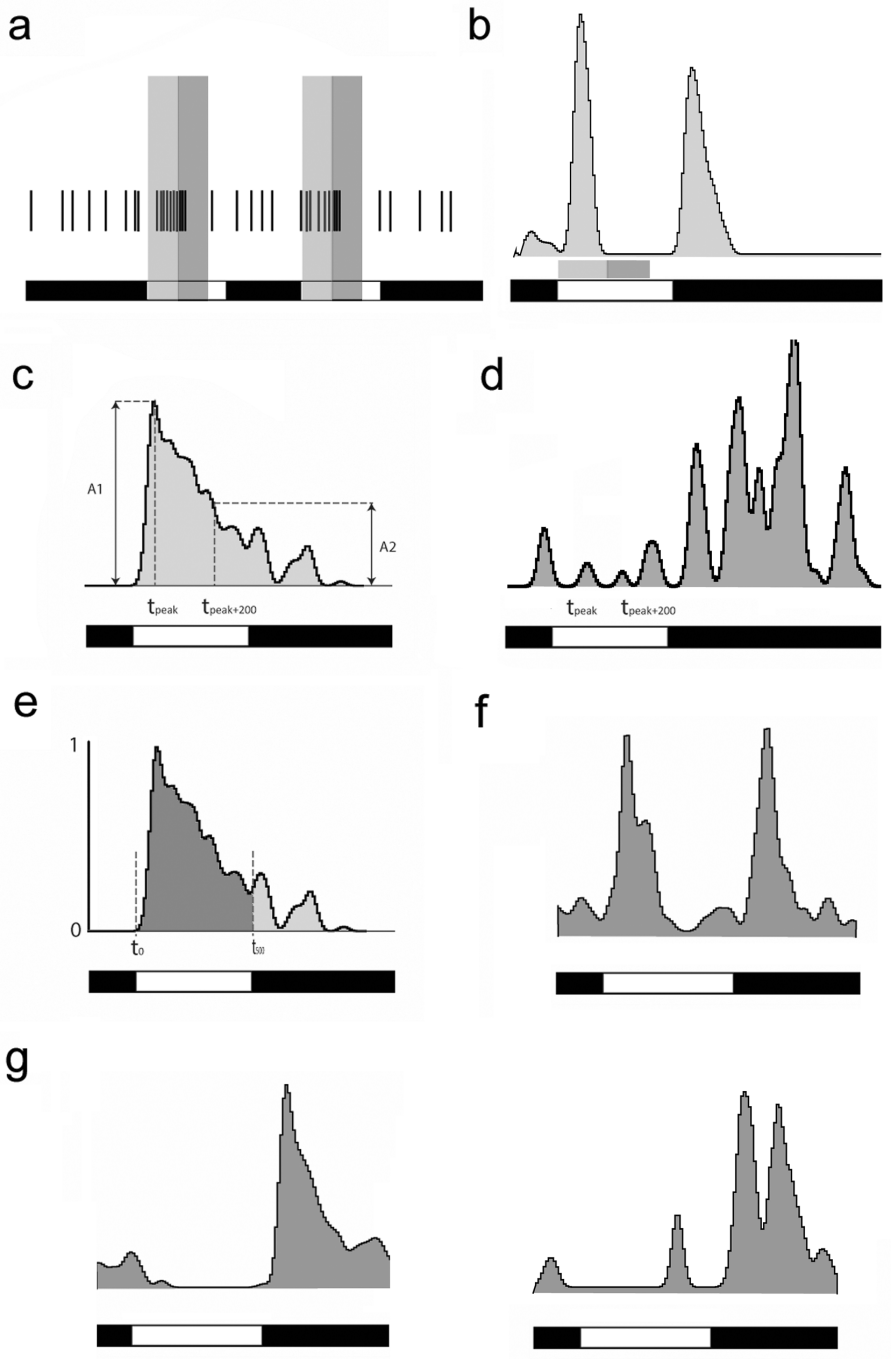
Previously used methods to determine transiency of RGC light responses. **(A).** modified Sustained-transience index (mSTI) is calculated by dividing the spikes (time stamps of spikes are shown on the top trace) that occur during the second 200 ms after the stimulus onset (dark grey shading) by the spikes corresponding to the first 200 ms of the response (light grey shading). **(B).** Panel displays the PSTH generated upon light responses of a representative ON-OFF RGC. In case of the ON response of this neuron no spikes are detected during the second 200 ms, thus calculated mSTI values (mSTI = 0) do not reflect response transiency properly. **(C).** The Tr_200_ measurement is performed by determining the exact time of the response peak (t_peak_) and then obtaining the amplitude value of the PSTH curve (A2) corresponding to the time that occurs 200 ms following the peak response (t_200_). **(D).** The Tr_200_ approach fails to provide a precise transiency value when the response PSTH displays fluctuations, local minimums and maximums. **(E).** The area measurement approach is performed by a normalization of the PSTH curve and then determining the area outlined by the PSTH curve and the on- (t_0_) and offsets (t_500_) of the light stimulus. **(F).** Contrary to the similar shape of ON- and OFF components of this ON-OFF RGC light response, the area measurement approach provides very different transiency values due to a more robust shoulder for the ON response component. **(G).** Two OFF RGC PSTHs resulted in similar area summation values, however they displayed very different response characteristics. The onset of the photopic light stimulus is shown by the white bar, whereas the black bar shows light offset in the bottom trace for all panels.

Throughout the nervous system neurons are divided into two populations based on their response temporal characteristics evoked by prolonged depolarizing stimuli. While some neurons adapt quickly to a sustained stimulus and provide a short burst of spikes as a response, other neurons maintain high frequency spiking throughout the stimulus duration. This latter phenomenon has been shown to be the result of the presence of active conductance in the cell membrane, such as the voltage-gated mediated potassium current in fast spiking interneurons [1]. Akin to the brain, RGCs in the retina maintain high frequency spiking as a response to prolonged stimulation or adapt quickly and produce a transient burst of spikes; such neurons are dubbed sustained and transient cells, respectively. Despite the fact that most RGC responses in the mouse display intermediary temporal features and thus fail to fall into discreet transient and sustained categories [2, 3], the above transient/sustained distinction has been widely used because transiency is an important feature to identify RGC subtypes. Moreover, transiency is also in a strong correlation with the function each RGC type plays in encrypting various aspects of the visual scenery: RGCs that detect the precise timing of visual events must generate transient responses in order to reliably detect repetitive events. On the other hand, RGCs encoding rather static features such as the luminance level in their receptive field serve their function better by providing elongated light responses. Many retinal neurons—like bipolar, horizontal and some amacrine cells—don’t generate action potentials, but their graded depolarization can still be characterized as either sustained or transient. While transiency, the speed of the response decay, is characterized by the response time constant (τ) for graded potential changes, in cells that produce action potentials it is determined by the length of the spike burst evoked by the stimulus instead [4, 5, 6, 7]. This discrepancy in the methodology impedes efforts to determine whether RGC response kinetics were inherited from presynaptic bipolar cells, due to active membrane conductance, mediated by precisely timed inhibition by inner retinal amacrine cells or resulted from the combination of these mechanisms. In addition, a number of response waveforms exist whose temporal characteristics are not described well via one or more previously utilized approaches (Fig 1; see descriptions below).

In this study we overview some already existing methods that have previously been utilized to quantify transiency of retinal RGCs and pinpoint some potential problems where they fail to predict response characteristics. Then, we show that a slight modification of a method first introduced by Zeck and Masland [2] results in an approach by which most of these obstacles can be resolved. In this approach the time constant (τ) is calculated upon peristimulus time histograms (PSTH) of RGC responses (PSTHτ approach). To prove its potency, we compare previous methods to this novel PSTHτ calculation under certain experimental paradigms. Additionally, we show that the PSTHτ method is compatible with τ measurements upon graded voltage recordings, thus it is ideal to compare RGC PSTHτ and τ values in order to examine how the RGC spike code is generated. RGC PSTHτ values can also be compared to graded presynaptic bipolar cell responses and therefore can be used to study the dynamics of signaling through parallel retinal pathways. Finally, we argue that regardless of the utilized method, there is a great variety in response temporal features and thus a clear distinction between sustained and transient RGC populations could not be made.

## Materials and methods

### Animals and preparation

Adult (P30–90) C57BL6 mice were deeply anesthetized with the inhalation of Forane (4%, 0.2 ml/l) and immediately after, they were sacrificed using cervical dislocation. The eyes and the retinas were removed under dim red illumination and hemisected anterior to the ora-serrata. Anterior optics and the vitreous humor were isolated and the resultant retina-eyecup was placed in a superfusion chamber that was mounted in a light-tight Faraday cage and perfused with an oxygenated and heated (34°C) mammalian Ringer solution (pH = 7.4). Animal handling, housing, and experimental procedures were reviewed and approved by the ethical committee of the University of Pécs (BA02/2000-6/2006; BA/35/51-42/2016). All animals were treated in accordance with the ARVO Statement for the Use of Animals in Ophthalmic and Vision Research. All efforts were made to minimize pain and discomfort during the experiments.

### Extracellular electrophysiology

Extracellular recordings were performed from retinal RGCs with carbon microelectrodes (1 MΩ; Kation Scientific LCC Minneapolis, MN, USA) and an AC differential amplifier (DAM80i, World Precision Instruments). All collected recordings were then digitized with an analog-to-digital board (Digidata 1440a; Axon Instruments, Sunnyvale, CA, USA) and further analyzed off-line. Single unit RGC action potentials were recorded digitally at a sampling rate of 20 kHz with Axoscope (Axon Instruments, Foster City, CA). Offline analysis was performed with Spike 2 (Cambridge Electronics Design Ltd., Cambridge, UK), Off-line Sorter (Plexon, Dallas, TX) and Neuroexplorer (Nex Technologies, Littleton, MA) software. Histograms and graphs were generated using Origin 7 (Microcal, Northampton, MA, USA). In order to compare transiency distribution functions obtained by the different approaches, we performed normalization on all data for the section ‘Transiency distribution of retinal RGC photopic responses’. This normalization was performed on the final transiency values separately for each methodological dataset. Responses of On-, Off- and On-Off RGCs were utilized in this study. Cells were considered to be On-Off if the two response components displayed about the same amplitude (amplitude of the dominant component was less than twice as high as the smaller response component).

### Intracellular electrophysiology and data analysis

Sharp electrode intracellular recordings were performed from retinal RGCs using microelectrodes prepared from borosilicate glass tubing with filament (1.2/0.6 mm for outer and inner diameters). Electrodes were filled 2 M KCl to produce a reversible junction with the Ag–AgCl connector. DC resistances of glass micropipettes ranged from 150–300 MΩ. All recordings were digitized with 20 KHz sampling frequency with an analog-to-digital board (Digidata 1320; Molecular Devices, Sunnyvale, CA, USA).

### Light stimulation

A green (l = 520 nm) LED was utilized that provided uniform full-field illumination on the surface of the preparation. In addition, well centered white light spot stimuli were also used for certain experiments (spot diameters: 10, 20, 40, 60, 80, 140, 180, 270 and 360 μm). In this case the stimulus was generated by a 100-W quartz-iodide lamp and intensity was adjusted with inserting calibrated neutral density filters in the light path. The timing of stimulus on- and off-sets and the intensity of square-wave impulses were controlled by a Master 8 pulse stimulator (A.M.P.I., Jerusalem, Israel). Light stimuli values were given in terms of the rate of photo-isomerization that occurs in each rod in every second (Rh*/rod/sec); we calculated with an average rod density of 437,000 rods/ mm2 [8] and quantum efficiency of 0.67 [9]. The intensity of the light stimuli varied from 1 to 6000 Rh*/rod/sec.

## Results

### Alternative methods used to determine transiency of RGC responses

Several various methods have been used to determine response transiency of RGC spike trains elicited by light stimulation. In some of these, checkerboard stimulation and RGC spike triggered average (STA) curves are used to obtain response transience [10], in others on the other hand peristimulus time histograms (PSTH) are generated upon stimulation and transiency values are derived via simple arithmetic calculations. In this study, these latter PSTH based methods are compared for their efficacy and reliability to describe temporal characteristics of RGC light responses.

Zhang and colleagues [11] generated sustained/transient indices (STI) by dividing the mean spike frequency during the second 400 ms by the mean spike frequency during the first 400 ms after the on- or offset of light stimulation for On and Off responses, respectively. Thereby, the STI value of a transient cell would be close to 0 while values of sustained cells converge to 1. Zhang and colleagues examined channelorhodopsin and halorhodopsin expressing cells where stimulus evoked responses had largely the same delays, thus the established STI values served well in that particular experimental design. Here we performed slight modifications on the STI calculations. This modified STI approach (mSTI) is depicted in Fig 1A, where the mean spike frequencies of the first and second 200 ms intervals were determined following the peak of the light evoked responses instead of following light on- or offset. This modification was necessary to compare the transiency of RGC responses in the mouse retina where response delays vary in a wide range. This method, however, fails to differentiate between responses where the second 200 ms time interval does not contain any spikes (Fig 1B), meaning that each response falling into this category would possess mSTI = 0 values regardless of the observable differences in their temporal characteristics.

Another way to quantify transiency was developed by Ganczer and colleagues (2016; Fig 1C). They generated peristimulus-time histograms (PSTH) upon RGC spike recordings and determined the delay (t_peak_) and amplitude (A_1_) of the histogram peak as well as the amplitude (A_2_) of the histogram 200 ms following the response peak (t_peak+200_). The transiency (Tr_200_) value was calculated simply by dividing the amplitude change (A_1_-A_2_) with the peak amplitude (Tr_200_ = (A_1_-A_2_)/A_1_), thus transient cells possessed high (~1) while sustained cells had low (~0) Tr_200_ values in a continuous range between 0 and 1. Unlike the previously described method (both STI and mSTI), the Tr_200_ approach provides a valid value for very transient responses as well, although it may give erroneous results when a PSTH displays a local minimum or maximum at around t_peak+200_ (Fig 1D).

A third alternative method was introduced by Farrow and Masland [3], in which transiency was calculated as the area under the normalized PSTH curve (area method). In this scheme, transient and sustained cells are characterized with low and high values, respectively (Fig 1E). Although this method is very sensitive and arguably more reliable than the previous approaches, it may offer ambiguous results under specific circumstances (Fig 1F and 1G). One such scenario occurs when a rather transient response with an elongated plateau phase and a sustained cell show similar values if area calculation was used to determine transiency.

**Fig 2:**
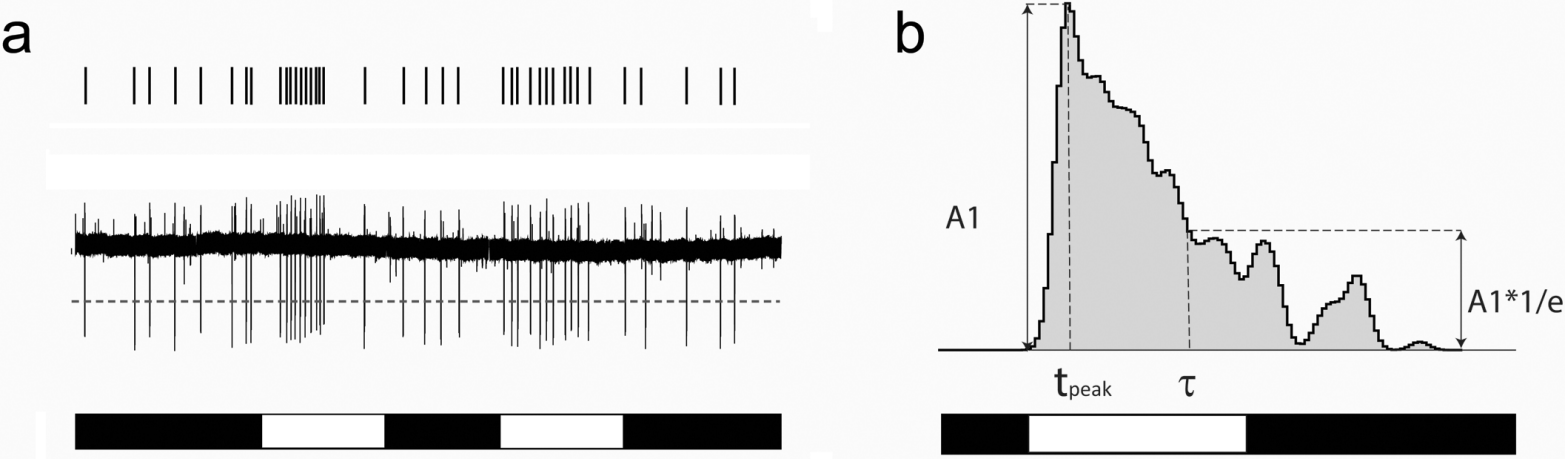
Obtaining PSTHτ transiency values in this study. **(A).** Extracellular spike recordings (middle trace) were detected upon photopic light stimuli (bottom trace). Spikes were sorted offline by using the appropriate threshold line (dashed line) to determine spike timestamps (top trace). **(B).** To quantify transiency, peristimulus time histograms were created upon light response timestamps using the light onset as reference. PSTHτ values were obtained by determining the peak time (t_peak_) and the peak amplitude (A1) and then A1*1/e was calculated and that gave the time constant itself (τ).

In this study, we adopt an approach used previously by Zeck and Masland [2] and modify it to determine the response transiency of retinal RGCs while avoiding most of the above mentioned limitations. In this scheme, first a PSTH is generated upon light responses (Fig 2A). Although a 10 ms bin size was utilized throughout this study, any bin size in the range of 10–50 ms works equally well. When smaller bins are utilized histograms depict finer temporal details of light responses but the result is also prone to trial-to-trial variability of responses. To overcome this issue we used a combination of small bins (10 ms) and averaging 6 consecutive light responses. Averaging of fewer than 4 responses may not eliminate most trial-to-trial variability while more than 8 light responses may induce the adaptation of the retina and result in corresponding changes in the responses. When the experimental design requires higher repetition numbers, the introduction of an extended pause is necessary between trials to avoid the aforementioned light adaptation process. In this study, we found that by using 6 consecutive light responses evoked by high photopic (I = 5000 Rh*/rod/sec), full-field, 500 ms long stimuli with 1.5 s inter-stimulus pauses did not cause any observable changes in the temporal characteristics of the individual light responses. Next, the precise time of the response peak (amplitude: A_1_) is determined to serve as time zero (t_0_) in further calculations. As a final step, the 1/e*A_1_ is calculated and the time constant (τ) of the PSTH curve is determined. Due to its analogy to time constants (τ) determined upon intracellular current or voltage recordings, this transiency value is coined as PSTHτ. The PSTHτ method provides transiency values even in specific cases when alternative methods fail (see above).

### Cross-trial reliability of the PSTHτ method

Next, we compared the performance of the PSTHτ method to those of the other approaches in distinguishing between transient and sustained responses of retinal RGCs. To this end an additional modification was performed for values of the Tr_200_ method. This was necessary because while mSTI, area calculation and PSTHτ provide lower values for transient and higher values for sustained responses, in Tr_200_ analysis high values refer to transient and low values to sustained responses. To avoid this inconsistency we subtracted all Tr_200_ values from 1, which resulted in a Tr_200_ value range similar in polarity to those of other approaches, therefore allowing comparison of all four methods.

**Fig 3:**
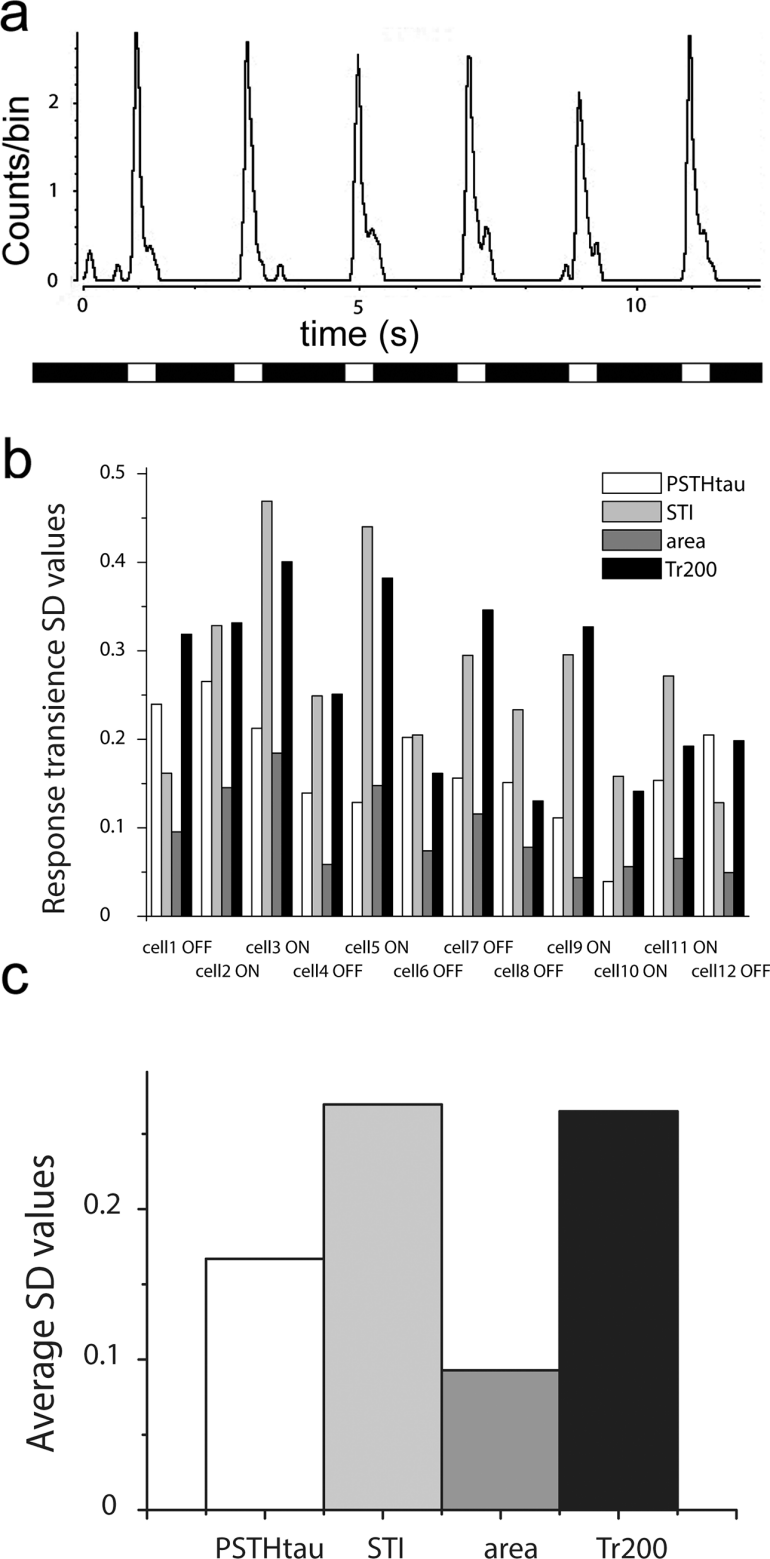
Trial-to-trial reliability of the PSTHτ approach. **(A).** Representative light responses of a mouse ON center cell evoked by six consecutive full-field, photopic light stimuli. **(B).** Although the same stimulus was presented to compare the reliability of the four different methods, the standard deviations (SD) for the calculated transiency values were very different when they were compared for twelve different cells. **(C).** Histogram showing values obtained by averaging the SD values for the four compared methods for RGCs from Fig 3B.

Our first goal was to compare the trial-to-trial reliability of the four methods. Therefore, RGC spike responses were recorded extracellularly upon photopic light stimulation (fullfield; λ = 480; I = 5000 Rh*/rod/sec). Six consecutive light responses were recorded for all retinal RGCs (n = 12; stimulus duration was 0.5 sec followed by 1.5 sec delay) and PSTHs were obtained for each individual response trial (Fig 3A). Transiency values as well as the trial-to-trial variations (standard deviation: SD) were calculated for the mSTI, the Tr_200_, the area and the PSTHτ methods. When SD values that represent variability across response trials were compared, it became clear that most of the time PSTHτ SDs were smaller or comparable to those of the alternative methods (Fig 3B). This indicated that, at least for our example cells, the PSTHτ method provided values that varied less across trials than most alternative methods. Thus, the PSTHτ appears to be a reliable approach for describing temporal characteristics of light responses. The average SDs of each method for the examined RGCs were also compared (Fig 3C). This comparison showed that in general area calculation and PSTHτ provided values with the lowest trial-to-trial variability, thus they are more reliable than the other two methods.

**Fig 4:**
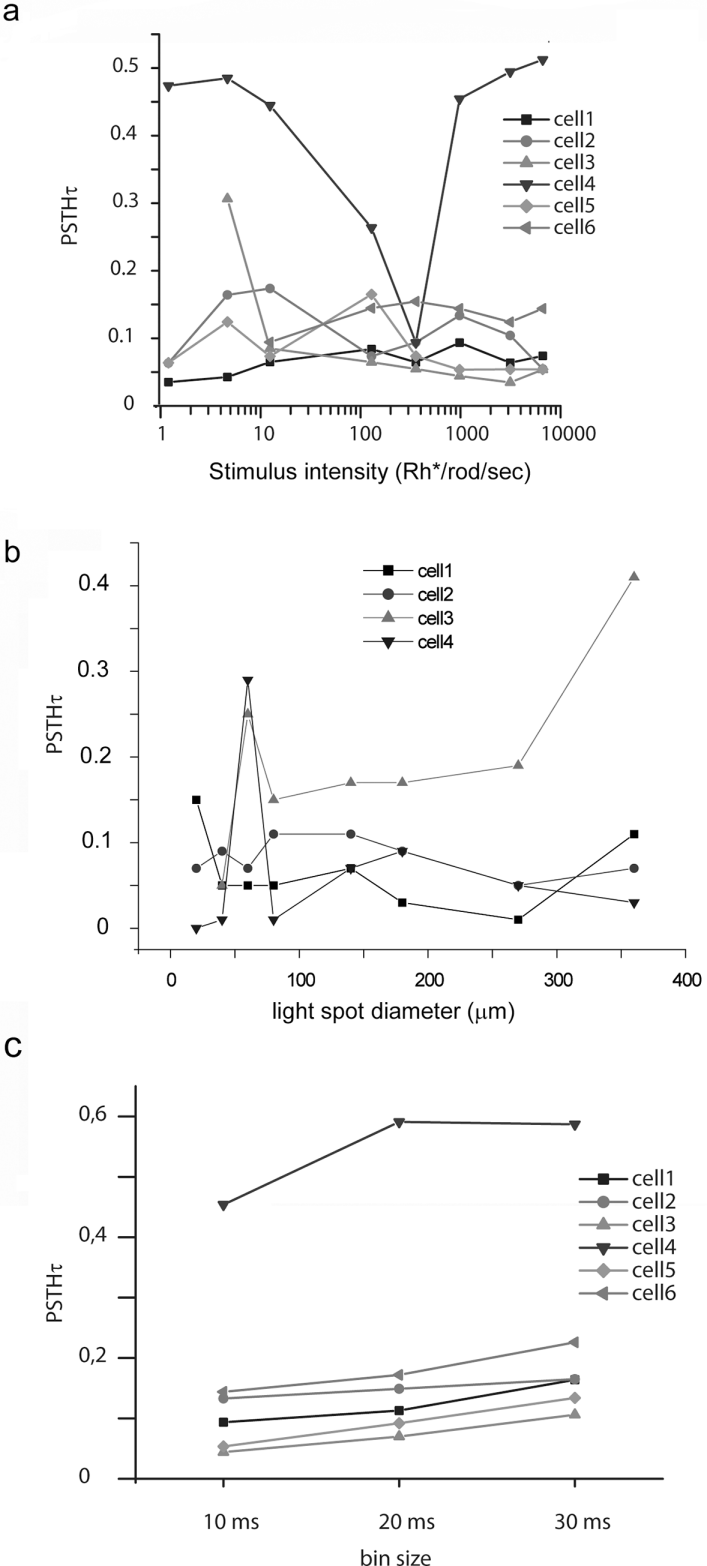
PSTHτ values depict the stimulus dependency of RGC responses. **(A).** Selected RGC (n = 6) PSTHτ values obtained upon full-field light evoked responses were stable (cells 1, 2, 5 and 6) while those of others (cells 3 and 4) displayed some intensity dependent change. **(B).** PSTHτ values of selected RGCs (n = 4) were obtained by a series of light spots with varying diameters (10, 20, 40, 60, 80, 140, 180, 270 and 360 μm). RGC PSTHτ values change according to the size of the presented stimulus. **(C).** Histogram is showing PSTHτ values for RGC (n = 6) responses. PSTHτ values were calculated for PSTHs obtained with different bin sizes (10, 20 and 30 ms) for each cell.

In addition to its reliability, the PSTHτ approach can also be utilized to examine the effects of stimulus parameters on transiency values. Fig 4 captures how these changes occur as a function of stimulus intensity and stimulus size. We found that some RGC PSTHτ values displayed a stimulus intensity (full-filled, λ = 542) dependent change (Fig 4A, cell4 and cell 3), whereas PSTHτ values for most RGCs remained largely unchanged throughout the entire intensity range used (1–10000 Rh*/rod/sec–scotopic to high photopic). This suggests that the stability of transiency throughout various stimulus intensities is a RGC subtype specific feature. PSTHτ values of selected RGCs (n = 4) were also tested by presenting a series of light spots with varying diameters. We found that RGC transiency displays some spot size dependent change (Fig 4B), as most examined RGC responses became more transient. This likely reflects the effect of RGC surround receptive fields that are activated only by larger stimuli. Altogether, PSTHτ values highly depend on stimulus parameters, therefore their values must be carefully selected for each experimental design. In addition, we also tested how methodological parameters, like selected bin size, alter PSTHτ measurements. Fig 4C shows that PSTHτ values gradually increased when the applied bin size was changed from 10 to 20 and to 30 ms. Although PSTHτ values somewhat depended on the selected bin size (generally increased), the overall temporal character was unaltered.

### Compatibility of PSTHτ to conventional slow potential time constant values

**Fig 5:**
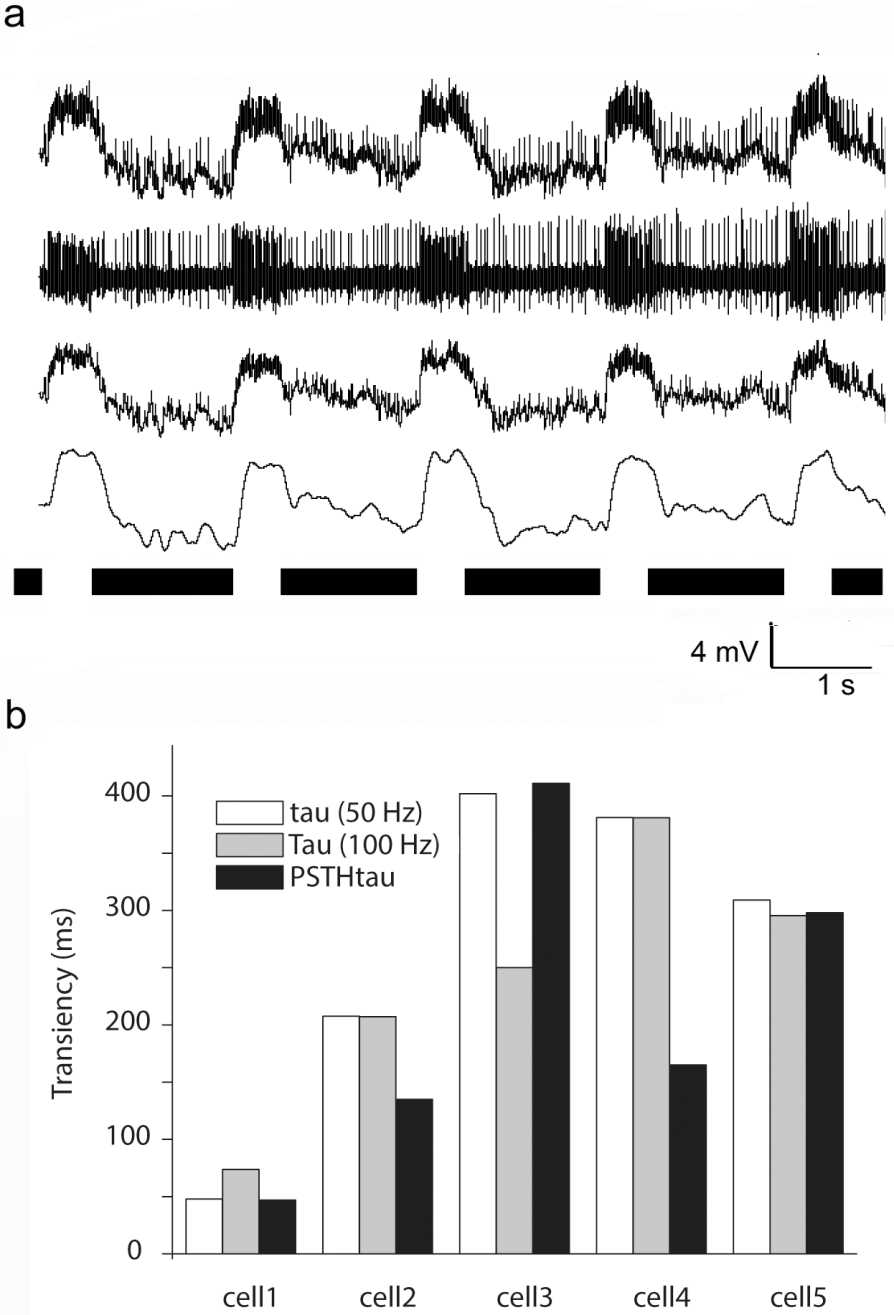
Comparison of PTSHτ and slow potential τ values. **(A).** Intracellular recording from an ON center RGC in the mouse retina that displays both slow potentials and spikes (upper trace). Spikes (second trace) and slow potentials (lower two traces) were obtained from the same recording by applying either a high-pass (>100 Hz) or a low-pass filter (<50 and 100 Hz for the third and fourth traces), respectively. Spike trains and slow potentials were then utilized to calculate both PTSHτ and τ values for the same light responses. **(B).** Bar-graph shows PTSHτ and τ triplets for randomly selected RGCs. In each column PTSHτ and τ values appeared comparable for the tested cells.

In order to test whether PSTHτ values are comparable to τ values generated upon RGC slow potentials, selected RGCs (n = 5) were recorded intracellularly to detect both slow potentials and spikes evoked by full-filled light stimulation for each examined cell (Fig 5A upper trace; λ = 480; I = 5000 Rh*/rod/sec). Recordings were first passed through either a 50 or a 100 Hz low-pass filter to obtain slow potential curves (Fig 5A third and fourth traces). Low-pass filtered curves did not contain spikes, allowing us to precisely determine individual τ values for each RGC response and to calculate average τ values for each RGC. The original unfiltered recordings were also passed through a high-pass filter (>100 Hz) in order to obtain the spike train for each of the recordings (Fig 5 second trace panel). This allowed us to generate PSTHs and to calculate PSTHτ values in each case. When τ and PSTHτ values were compared we found that they displayed comparable values, (Fig 5B) providing further evidence that the PSTHτ method describes the temporal characteristics of retinal RGC responses. In addition, this latter analysis also allowed us to examine the relation of slow potential temporal characteristics to those of light evoked spike bursts. Interestingly, we found that whereas a PSTH and a slow potential obtained from a certain RGC response displayed similar shapes and thus yielded comparable τ and PSTHτ values, their delays sometimes differed. Very typically we found that PSTH peaks displayed a shorter delay than the corresponding slow potentials (not shown). This observation suggested that the maximum frequency of spike bursts occurred when slow potentials were in the rising phase and not when they reached their maximum.

### Transiency distribution of retinal RGC photopic responses

**Fig 6:**
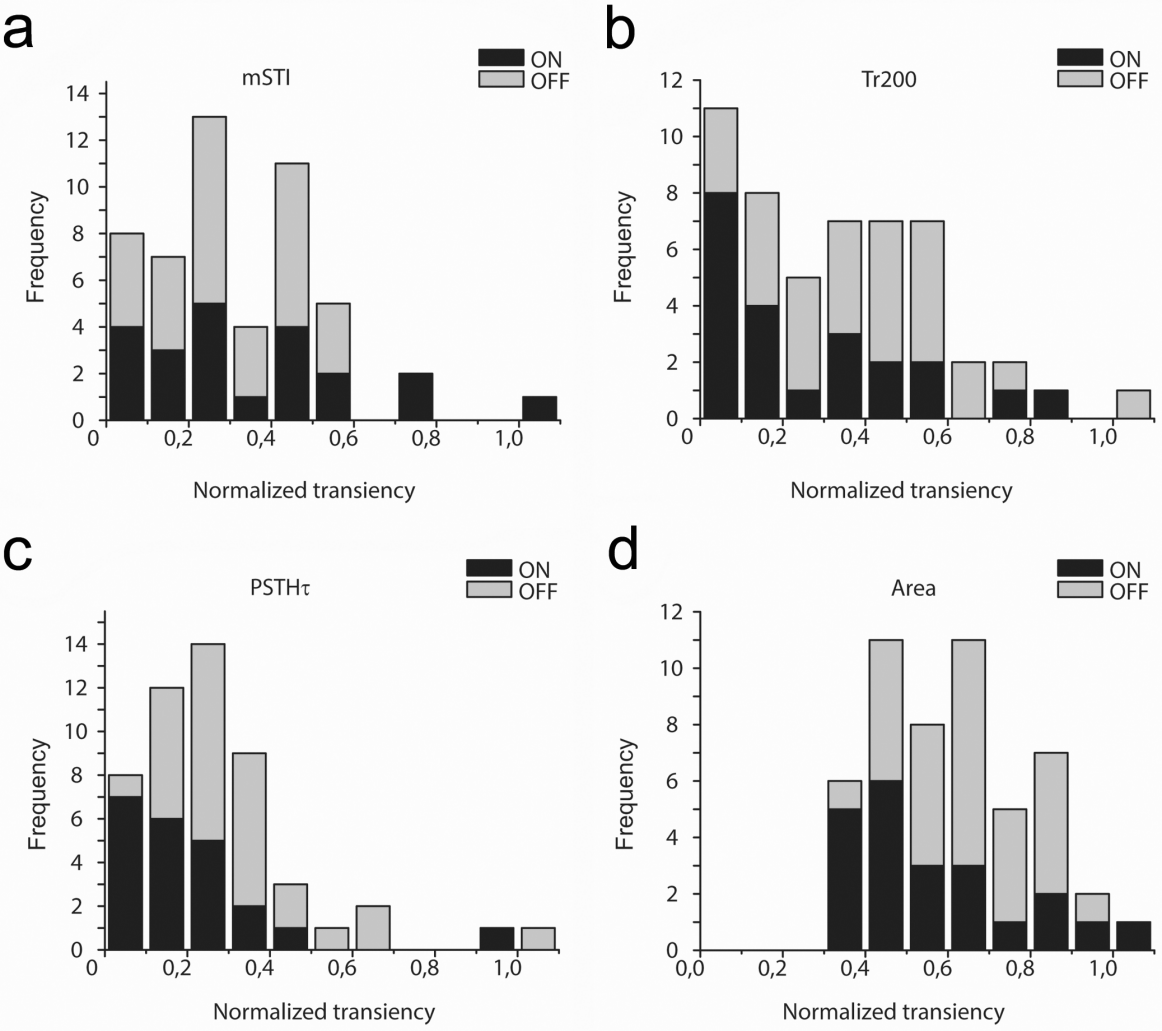
Transiency of RGC responses failed to form two distinct subpopulations corresponding to sustained and transient cells. **(A-D).** Frequency histograms show the distribution of RGC (n = 51) response transiency values for all four alternative methods. Clearly, none of the frequency histograms appear to display two well separable populations of RGC responses that correspond to sustained and transient subgroups of RGCs. The obtained RGC transiency values were rather distributed randomly (mSTI) or in a quasi-lognormal fashion (PTSHτ and area calculation).

One long-term purpose of obtaining transiency values for neuron responses is to categorize them and differentiate between distinct physiological classes. Retinal neuron responses have previously been described either as sustained or transient with the utilization of the above listed methods [3, 11]. While some RGCs fell into either into the transient or the sustained category, most of them displayed intermediary response temporal features [2, 3]. Here we tested whether any of the four above mentioned approaches could be used to sort RGC responses into clearly distinguishable transient and sustained categories. Using uniform full-field photopic (I = 3000 Rh*/rod/sec) light stimulation, transiency values were collected from a cohort of RGCs (n = 51; n = 15 mice) using each of the previously described methods. Responses of On- and Off-cells as well as both response components of On-Off RGCs were utilized. In order to gain a comparable dataset, all obtained values were normalized (note that original mSTI, Tr_200_, area and PSTHτ values are distributed on different scales). Distribution histograms then were generated upon the obtained dataset (Fig 6). Interestingly, distribution histograms that were generated upon values of the same set of RGC responses displayed very different results for the four methods: (i) only the values of Tr_200_ data covered the entire range (0 to 1), (ii) the transiency distribution histograms generated for Off responses fell into a more sustained domain than those of the On responses for the Tr_200_, PSTHτ and area measurements and (iii) mSTI, Tr_200_ and PSTHτ histograms were skewed to lower values whereas the area histogram was not. Despite these differences in the distribution of the four populations of transiency values, the histograms shared one common feature: none of them displayed a clear presence of two well-separable response subpopulations. The mSTI and Tr_200_ methods appeared to show such division of suspected response populations at value ~0.3, however, this apparent distinction only occurred for On- but not Off responses. Overall we found that the temporal characteristics of RGC responses did not follow the transient/sustained dichotomy but rather, they formed a broad continuum. This suggests that regardless of the methodology, only a fraction of RGCs produce clear transient or sustained responses, most of the examined RGCs appear to display an intermediary response character.

## Discussion

### The suitability of PSTHτ to describe temporal characteristics of retinal RGCs

According to Anishchenko and colleagues [10] transience of some rat RGC responses are not fully mature at the time of eye opening, therefore one may argue that age dependent changes in response kinetics interfere with our findings. In this study P30-P90 mice were utilized for all experiments. Considering that the final important steps in brain development of mice occur by P12 [12] and menstruation typically begins between postnatal day 25 and 40 we consider all mice utilized in this study as young adults. However, regardless the age of the mice all comparisons were performed between analyzing methods and not between responses obtained from different animals, therefore the age of the utilized animals is an indifferent factor in our present work. By comparing transiency values obtained from the above described and previously used three methods we found that each approach possesses weaknesses that could provide misleading results in certain cases. Therefore, the purpose of the present study was to compensate for most of these shortcomings and establish a simple, practical and reliable way to measure and quantify transiency, the temporal response characteristic of RGC light responses. To this end we utilized the PSTHτ method that provides τ values (1/e of maximum) for PSTH histograms generated upon light responses. This is analogous to the time constat (τ) that is determined for intracellular current and potential recordings. A comparison of transiency values obtained with the tested approaches indicated that each method had advantages over the others in certain circumstances (Table 1). The mSTI and area calculations for example give sum values of many bins thus they are less prone to local variability in PSTH curves. On the other hand, however, they are not capable of providing any information about the shape of PSTH curves for the exact same reason. When trial-to-trial reliability is compared both area measurement and PSTHτ over-perform the other two approaches but at the same time area calculation fails to compensate for differences in response delay and its values are not comparable to simultaneously measured local potentials. Therefore, we think that the tested methods are largely complementary, and among them the overall performance of the PSTHτ approach appears to be the most favorable. In our tests, while remaining susceptible in certain cases to the appearance of local minimums, PSTHτ provided a reliable trial-to-trial similarity (low SD values) for repeated light stimuli, reflected the actual shape of the light response curve, compensated for response delay and was compatible with local potentials. This latter feature could be very important in experimental protocols when signaling between presynaptic non-spiking and postsynaptic spiking neurons are examined. For example, signaling throughout the retinal neuronal network could be studied by simultaneously monitoring local potential changes detected in presynaptic bipolar cells and spike trains of postsynaptic RGCs both evoked by the same light stimuli. In this scheme, transiency of bipolar cell responses can be determined by calculating τ values (Ichinose 2016) upon local potential changes (or currents), whereas PSTHτ values can refer to RGC response transiency. A similar comparison of non-spiking presynaptic bipolar cell and postsynaptic spiking RGC responses with the other alternative methods would not be feasible.

**Table 1:** Comparison of alternative methods to characterize response transiency regarding various criteria. Symbols +, ++ and +++ represent weak, good and excellent performance.

Method	Reliability (trial-to-trial variation)	Shape	Resistance to local variability	Capability to differentiate between populations	Compensation for response delay	Comparability to slow potentials
mSTI	+	+	+++	++	+++	+
Tr200	++	++	+	++	+++	+++
area	+++	+	+++	+	+	+
PSTHτ	+++	+++	++	+	+++	+++

### Transient-sustained continuum of RGC responses

Despite the relative good performance of the PSTHτ method in most of our analyses, it failed to clearly separate a sustained and a transient RGC response population. However, all other alternative approaches failed to distinguish clearly between these two RGC response populations as well. This finding strongly suggests that RGC responses in general fail to form two distinct populations, and instead, they rather all belong to a single wide continuum regardless of the utilized approach. This is very consistent with previous reports [2, 3]), where mouse RGC responses also failed to fall into discreet transient and sustained categories. While these studies, including this present work, were restricted to mouse RGC light response recordings, it is possible that responses from other vertebrate species would result in a better transient-sustained response separation. Regarding the transience continuum of mouse RGC responses it is unclear whether it is due to a response variability of individual RGCs in each RGC subtype (in-class variability) or a consequence of response variability across various RGC classes. While in-class variability of RGC responses exists, it has been shown that certain RGC classes can be distinguished by means of the temporal features of their responses including transiency [13, 14]. Therefore, the observed response continuum must be the consequence of response variability across RGC classes. The above finding on the continuum of mouse RGC response transience, however, is somewhat surprising because a clear transient-sustained dichotomy have been found in both mammals and lower vertebrates for retinal bipolar cells that provide the main excitatory input for RGCs [4, 5]. Major differences in bipolar cell response temporal characteristics were attributed to the different kinetics of glutamate receptors at the photoreceptor-to-bipolar cell synapses [5] as well as the different transmitter environments that invagination and flat contacts provide [15]. In addition to the photoreceptor-to-bipolar cell synapse, other downstream factors may affect bipolar cell response transiency as well including the presence of active conductance and feed-back inhibition [16, 17, 18]. However, regardless the origin of bipolar cell transiency, RGCs receive much of their inputs from these same bipolar cells, therefore one may expect that RGC responses inherit their temporal characteristics from presynaptic bipolar cells and show little further change. The discrepancy in the temporal characteristics of RGCs and retinal bipolar cell responses could be explained by the fact that each RGC receives inputs from several bipolar cell types that co-stratify (at least partially) with their dendritic arbors. A mixed bipolar cell input to postsynaptic RGCs could thus contribute to the generation of intermediary (in-between sustained and transient) responses. Of course, RGCs receive inputs of non-bipolar cell origin as well, including inhibitory inputs from amacrine cells and excitatory electrical synaptic inputs from electrically coupled amacrine and/or RGC neighbors [19, 20, 21, 22, 23, 24]. All these additional inputs may alter RGC response characteristics. For example, inhibition may truncate GC response PSTH peaks or make it more transient depending on the precise timing of the given inhibition. Similarly, excitation from neighboring gap junction coupled neurons can make RGC responses more sustained or more transient in a timing dependent manner. In addition to various inputs, the active and passive membrane properties can also influence the temporal characteristics of RGC responses as well [25]. Thus, all the above mentioned mechanisms likely contribute to the temporal characteristics of RGC responses. As a result, when RGC responses are examined across the entire population, they cannot be sorted into two well separable sustained and transient populations but rather, they appear to form a continuum. For that reason, describing RGC responses either as sustained or transient appears to be an oversimplified and insufficient characterization scheme. Therefore, we think that a useful approach should characterize RGC responses with values on a continuous scale and the previously described PSTHτ method is suitable for this function.

### Compatibility of transience measurements of retinal neurons

Photoreceptors respond with membrane potential hyperpolarization to increments in light intensity. Although the response amplitude is in a close correlation with the light intensity change, the transiency of photoreceptor responses varies little. Bipolar cells also respond with graded membrane potential changes to stimuli while the polarity and the transiency of their responses display a subtype dependent variety, which is highly regulated by the kinetics of the postsynaptic glutamate receptors they express [4, 5, 15]. Upon glutamate binding, bipolar cells produce graded membrane potential changes whose temporal characteristics can be readily described by a sole value, τ, that reflects the speed of response decline. Besides graded potentials, RGCs also generate action potentials upon stimuli and the signal they transmit to the brain is encoded by the rate of action potentials [26]. In order to describe signaling throughout the early visual system, it is necessary to provide a characterization scheme in which transiency values from photoreceptors through bipolar cells to RGCs can be compared regardless of the encoding mechanism (graded potentials or spike trains) of encompassing neurons. The PSTHτ method fulfills this requirement by providing a measure to describe the decline in the RGC spiking rate akin to the τ value obtained for RGC slow potentials. Although parallel bipolar/ganglion cell recordings were not performed in this study, the similar nature of RGC PSTHτ and presynaptic bipolar cell τ values provide a basis for comparison of their responses to each other and also to other neurons along the visual axis. In addition, the PSTHτ method offers an opportunity to examine the way RGC graded potential changes are translated into spike trains. Whereas certain neurons provide spike trains that reach maximum frequencies (PSTH peaks) near the corresponding depolarization peaks, other neurons display faster spiking kinetics that result in a discrepancy between the depolarizations and the PSTH peaks. Details about light responses including the time and amplitude of peak spiking rate are not depicted by mSTI and area calculation methods (as they were not designed to do so), thus they could not be utilized for such observations. In contrast, when data are collected for the PSTHτ approach these details are directly measured and can be utilized for further analysis.
